# Transcriptional profiling of *Chlamydia trachomatis* and its host in an *ex vivo* endocervical primary cell culture system using dual RNA sequencing

**DOI:** 10.3389/fcimb.2025.1613922

**Published:** 2025-06-17

**Authors:** Olusola Olagoke, Siddharth Chittaranjan, Deborah Dean

**Affiliations:** ^1^ Departments of Medicine and Pediatrics, University of California, San Francisco, Oakland, CA, United States; ^2^ Department of Bioengineering, University of California, San Francisco, San Francisco, CA, United States; ^3^ Department of Bioengineering, University of California, Berkeley, Berkeley, CA, United States; ^4^ Bixby Center for Global Reproductive Health, University of California, San Francisco, San Francisco, CA, United States; ^5^ Benioff Center for Microbiome Medicine, University of California, San Francisco, San Francisco, CA, United States

**Keywords:** *Chlamydia trachomatis*, dual RNA sequencing, primary endocervical stromal cells, chlamydial pathogenesis, host pathogen environment interactions, bioinformatics

## Abstract

*Chlamydia trachomatis* (*Ct*) is an obligate intracellular bacterium that causes significant ocular and urogenital morbidity worldwide. Understanding host-pathogen interactions is challenging but dual RNA sequencing offers simultaneous transcriptome data for comprehensive interrogations into these interactions. While transcriptional profiling of both *Ct* and host-derived immortalized cells has been performed, this study used dual RNA sequencing to examine host-pathogen interactions in *ex vivo* human primary endocervical stromal cells infected with *Ct* strain E/Bour. At 1-hour post-infection (1hpi), 168 differentially expressed host genes (DEGs) were identified, 40% of which were non-coding RNAs, novel proteins, or pseudogenes. Pathway analysis revealed no significant enrichment at this stage, indicating a quiescent host response. At 24hpi, 212 DEGs were identified, with strong upregulation of interferon-stimulated genes and activation of the cGAS-STING and RLR pathways, despite the absence of detectable type I interferons. Pro-inflammatory and leukocyte recruitment genes were also highly expressed, suggesting an immunoreactive phenotype at this later stage. *Ct* transcriptomics identified 331 early and 903 mid-infection genes. Inclusion-membrane genes peaked at 1hpi, while hemolysin-like and polymorphic membrane protein genes were upregulated at 24hpi. Enrichment analysis identified pathways related to catalytic activity, host modulation, and bacterial survival. This study demonstrates distinct temporal dynamics in *Ct*-host interactions, including early host immune quiescence and robust mid-infection activation of innate immunity in contradistinction to previous host and *Ct* findings in immortalized cell lines. The findings emphasize the utility of *ex vivo* human primary cell culture for investigating *Ct* pathogenesis using clinically relevant *Ct* strains and provide a foundation for future exploration of uncharacterized genes and pathways critical to *Ct* infection.

## Introduction

*Chlamydia trachomatis* (*Ct*) is an obligate intracellular bacterium and the leading cause of sexually transmitted infections (STI) worldwide (Sexually transmitted infections (STIs)). *Ct* STIs are frequently asymptomatic, and if left untreated in the female genital tract, can lead to pelvic inflammatory disease, ectopic pregnancy, and infertility (Land et al., 2010; Rowley et al., 2019; Sexually Transmitted Disease Surveillance, 2023). *Ct* exhibits a biphasic developmental cycle that alternates between an infectious, non-replicative elementary body (EB) and a replicative, metabolically active reticulate body (RB). However, if RBs are exposed to host environmental stressors such as antibiotics, RBs can halt replication and enter a reversible persistent state that prioritizes cell functions required for long-term survival (Panzetta et al., 2018).

Research on the dynamics of *Ct* gene expression have increasingly substantiated how *Ct* modulates host-pathogen interactions that drive pathogenesis. Time course experiments have previously identified transcriptional changes throughout *Ct* development, categorizing them into temporal groups: Early (1–3 hours) to mid-early (>3–8 hours), mid (>8–24 hours), and late (>24 hours) gene expression (Belland et al., 2003b; Nicholson et al., 2003; Wurihan et al., 2024). One *in vitro* modelling study evaluated gene activity during persistence by monitoring *Ct* transcriptional responses to interferon (IFN)γ and identified key pathways that were differentially regulated (Belland et al., 2003a). For instance, *Ct* genes involved in phospholipid utilization and protein translation were found to be upregulated (Belland et al., 2003a). A rigorous understanding of the mechanisms that maintain and regulate the *Ct* transcriptome within the context of the host response can greatly enhance the development of diagnostic tools and preventive strategies for *Ct*.

Dual RNA-sequencing (dual RNA-seq) provides the simultaneous interrogation of both the host and pathogen transcriptomes (Westermann et al., 2017). Despite the potential of this technology, its application in chlamydial pathogenesis has thus far been limited to the study of *Ct* infection in immortalized cells (Humphrys et al., 2013; Hayward et al., 2021). Immortalized cells are unlikely to represent the actual *in vivo* response to pathogen infection. Building on our previous study of the human primary endocervical cell model of *Ct* infection, we found that human primary endocervical stromal (ECS) cells, unlike primary endocervical epithelial cells, elicited a variety of responses including pro- and anti-inflammatory mediators that varied depending on the infecting *Ct* ocular or urogenital strains (Jolly et al., 2019). We therefore applied dual RNA-seq to investigate the host-pathogen dynamics of *Ct* infection of ECS cells with urogenital reference strain E/Bour and mock infection of ECS at the early (i.e., 1 hour post infection [1hpi]) and mid (24hpi) timepoints of development. In the present study, we found unique host-pathogen responses compared to the literature that can be used to further elucidate *Ct* pathogenic mechanisms.

## Materials and methods

### *C. trachomatis* infection of primary cells

ECS cells were prepared from biopsied cancer-free de-identified hysterectomy tissue as previously described (Jolly et al., 2019). Briefly, tissue plugs from a single donor were plated the same day as the tissue biopsy on fresh culture dishes, grown in RPMI 1640 + 10% FBS Media, and fibroblasts were collected 1 to 2 weeks later, as they migrated from the tissue plug during this period. Confirmation of the cell type was based on fibronectin-specific antibody staining (Sheep anti-human fibronectin antibody, R&D systems; Pittsburgh, PA) of the cells. ECS cells used in this experiment were at passage number 2-3. ECS cells were plated in two 6-well tissue culture dishes (9.6 cm^2^ per well) in RPMI 1640 + 10% FBS Media (Lonza, Allendale, New Jersey) in the absence of antibiotics/antifungal agents. One plate contained two triplicate experiments for each condition (infected and mock-infected) at one timepoint. The timepoints of 1hpi and 24hpi were tested. Cells were grown to 80% confluency as confirmed by brightfield microscopy and then used to calculate the volume of inoculum required to achieve the target multiplicity of infection (MOI) of 1. The cells were mock-infected or infected with *Ct* reference strain E/Bour to achieve an MOI of 1 and centrifuged for 1 hour at 1500 rpm, 37°C, to facilitate bacterial contact with the cells. The infected cells were further incubated for one hour or 24 hours at 37°C with 5% CO_2_. At 24hpi, immediately prior to harvesting, the cells were visualized using a Nikon T1-E real time microscope with 5% CO_2_ under high-resolution imaging to confirm an MOI of 1 similar to our previous study (Jolly et al., 2019). Cells were washed twice with DPBS (ThermoFisher, Waltham, MA), harvested in TRI reagent (Zymo Research Corp, Irvine, CA) and immediately subjected to RNA extraction.

### Total RNA isolation, rRNA and polyA depletion, and library preparation and sequencing

Total RNA was extracted with Zymo Research Direct-zol kit (Zymo Research Corp, Irvine CA) following manufacturer’s instructions. RNA samples were frozen in -80°C for storage and then transferred to the sequencing facility (Functional Genomics Laboratory, UC Berkeley, Berkeley, CA). RNA concentrations of total RNA samples were measured using Qubit™ RNA HS Assay Kit (Invitrogen; Carlsbad, CA), and RNA integrity was measured using a Bioanalyzer 2100 Expert and the Agilent RNA 6000 Pico assay (Santa Clara, CA). All samples had an RNA integrity number greater than 7.0. To enrich samples for mRNA, all total RNA samples underwent ribosomal RNA depletion using Illumina Human and bacteria Ribo-Zero rRNA Removal Kits (Human/Mouse/Rat, and Bacteria; San Diego, CA). At this point, rRNA depleted RNA from mock-infected samples were sent for the library preparation phase of the procedure. rRNA depleted RNA from infected samples were split into two portions. One portion was sent directly for the library preparation phase of the procedure while the other portion was enriched for prokaryotic mRNA using the KAPA mRNA Capture Kit (Roche, San Francisco, CA) prior to sequencing. Illumina mRNA sequencing libraries were prepared using the KAPA mRNA HyperPrep Kit (Roche Sequencing Solutions, Pleasanton, CA) as per manufacturer’s instructions. Prior to sequencing, the quality of the libraries was verified on an AATI Fragment Analyzer (Agilent). Individually indexed RNA libraries were pooled and sequenced with Illumina NovaSeq 6000 using 150 bp paired-end reads, yielding an approximate 10 Gb per sample. Sequencing data were demultiplexed and analyzed independently. A simplified workflow from RNA extraction to sequencing is shown in **Supplementary Figure S1**.

### RNA sequence read processing, quantification and gene expression analysis

Host transcript quantification was performed using the Nextflow rnaseq package (Patel et al., 2024) with default settings. To quantify the bacterial reads, quality control on sequenced reads was performed using Fastp (Chen, 2023). Reads were subsequently depleted of contaminating host reads using Scrubby (Steinig, 2024). With Scrubby, two host read removal methods were implemented: Kraken2, with taxa set as Chordata, and Minimap2 targeting the human genome (GRCh38). Host-depleted reads were subsequently run through the Nextflow rnaseq package (Patel et al., 2024) for read quantification using the *Ct* E/bour genome assembly (NC_020971.1 and NC_020947.1) as reference genome. *Ct* E/bour gene identifiers were compared and converted to *Ct* D/UW-3/CX gene identifiers where necessary.

Differential gene expression analysis of host and bacterial reads was performed using the DESeq2 (Love et al., 2014) package on the iDEP (Ge et al., 2018) online platform with default settings. This included normalization to account for differences in library size across all samples. Genes with zero counts across all samples were filtered prior to analysis. To select differentially expressed genes for downstream analysis, a cut-off of a false discovery rate (FDR) adjusted p-values of ≤ 0.05 and log_2_FC ≥ 2 was used, except otherwise stated. To identify enriched biological pathways, a reactome analysis was performed using gProfiler (Raudvere et al., 2019), STRING (Szklarczyk et al., 2018) and ShinyGO (Ge et al., 2020).

## Results

### Host transcriptome of primary cells during *C. trachomatis* infection

**Figure 1:**
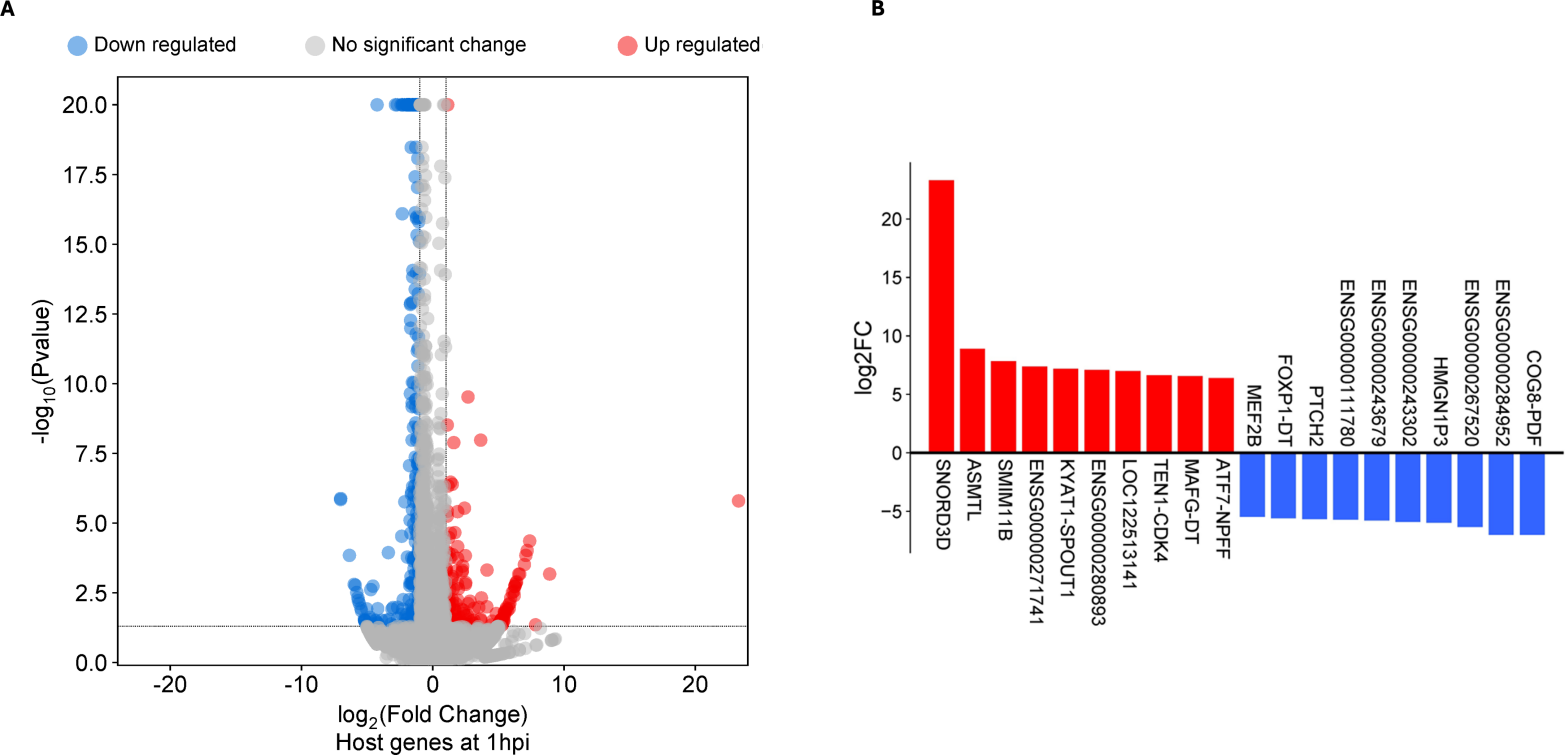
Differentially expressed genes (DEGs) in human primary endocervical stromal (ECS) cells infected with *C*. *trachomatis* reference strain E/Bour relative to mock infected cells at 1 hour post infection (1hpi). **(A)** Volcano plot showing differentially expressed genes (DEGs) at 1hpi (FDR ≤0.05, log_2_FC ≥2.0). **(B)** Top DEGs (n=20) at 1hpi.

Differentially expressed genes (DEGs) were defined as genes meeting the minimum cut-off of log_2_ fold change (LFC) ≥2 and a false discovery rate FDR of ≤0.05 when mock-infected cells were compared to *Ct* reference strain E/Bour infected cells. Using this threshold, 168 genes were found to be differentially expressed in ECS cells at 1hpi: 107 genes were upregulated, while 61 genes were downregulated (**Figure 1a**; **Supplementary Table S1a**). At an LFC of 23, Small Nucleolar RNA C/D Box 3D (SNORD3D) was the highest upregulated gene. Other top upregulated genes include Acetylserotonin N-Methyltransferase-Like (ASMTL), Small Integral Membrane Protein 11B (SMIM11B) gene and ENSG00000271741, a novel transcript of ZMYM6-ZMYM6NB readthrough. Top downregulated genes include those encoding a novel protein gene (ENSG00000272617) and a novel transcript (ENSG00000284952) in addition to a long non-coding RNA (ENSG00000267520) (**Figure 1b**). A substantial number of DEGs identified at 1hpi (n=68, ~40%) were either long intergenic non-protein coding RNAs (lincRNA), novel protein genes and transcripts, or pseudogenes (**Supplementary Table S1a**).

Gene set enrichment analysis of the host reactome was performed on the DEGs present at 1hpi. For this analysis, pathways with an FDR ≤0.05 were considered significant. This analysis identified no significantly enriched pathway at 1hpi. To assess whether our strict log_2_FC ≥2.0 for DEGs requirement limited downstream interpretation, we relaxed the cutoff slightly down to 1.5. Re-analysis showed that there was a significant downregulation of signaling especially by Rho GTPases, Miro GTPases and RHOBTB3 (**Supplementary Table S2**), suggesting that suppression of intracellular and cell-to-cell signaling was crucial to entry and establishment of *Ct* infection in ECS cells. Genes involved in these pathways include ARHGAP11B, HNRNPC, PPP2R5D, ARHGEF11, RHOBTB3 and CDC42EP3. A similar re-analysis of upregulated genes at 1hpi failed to yield any enriched pathways. This lack of significantly enriched pathways may be due to the abundance of long intergenic non-protein coding RNA, novel proteins and transcripts, or pseudogenes (**Supplementary Table S1a**).

**Figure 2:**
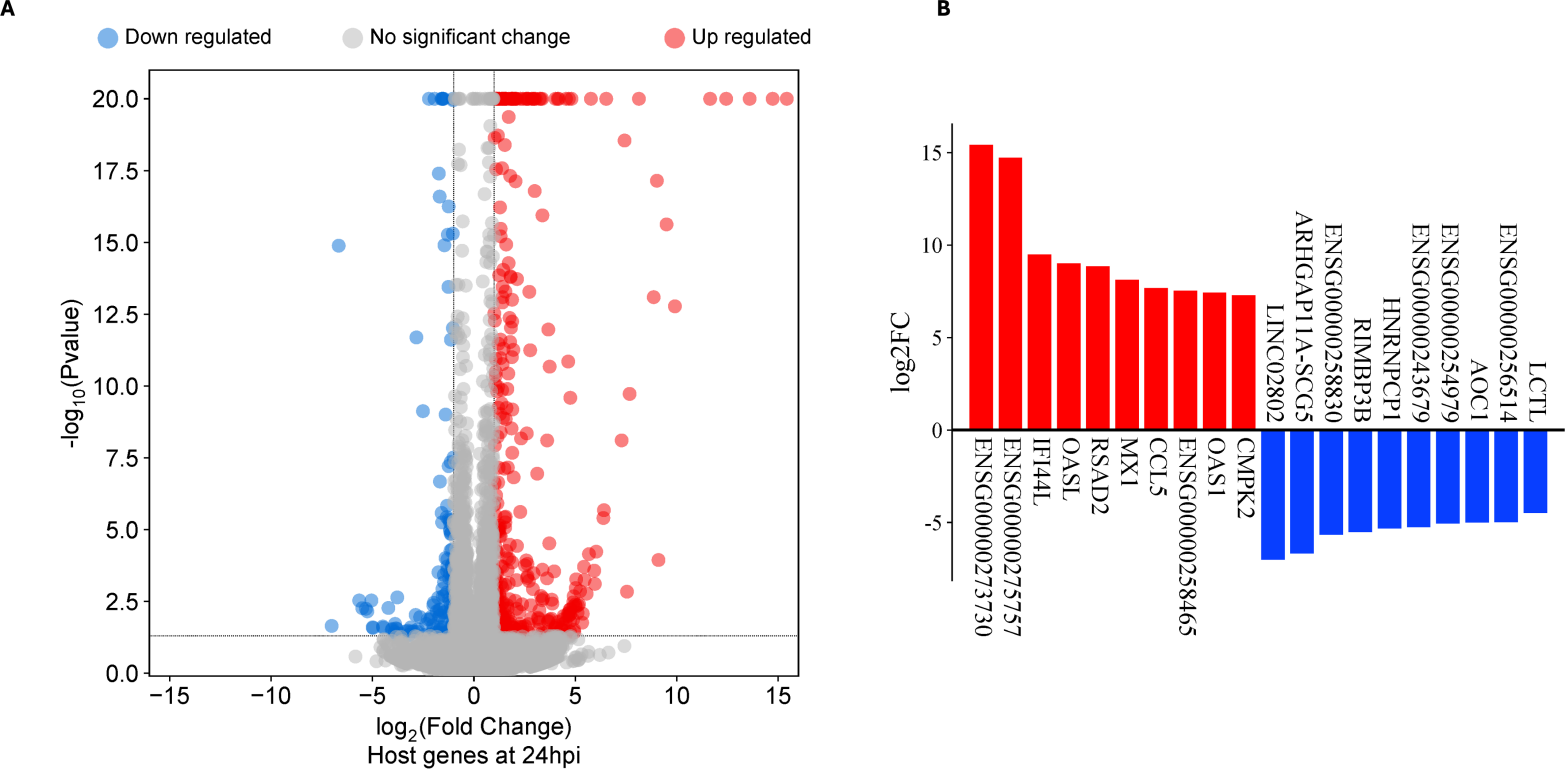
Differentially expressed genes (DEG) in human primary endocervical stromal (ECS) cells infected with *C*. *trachomatis* (*Ct*) reference strain E/Bour relative to mock infected cells at 24 hours post infection (24hpi). **(A)** Volcano plot showing DEGs in *Ct* E/Bour strain infected ECS cells at 24hpi (FDR ≤0.05, log_2_FC ≥2.0). **(B)** Top DEGs (n=20) in *Ct* infected ECS cells at 24hpi.

At 24hpi, 212 DEGs were identified, of which 164 genes were upregulated while 48 genes were downregulated (**Figure 2a**; **Supplementary Table S1b**). Six of the top 10 upregulated DEGs at 24hpi were IFN stimulated genes participating in the type I IFN pathway (**Figure 1b**). When we compared all DEGs at 1hpi and 24hpi, only eight were present at both timepoints, and seven of them had similar magnitudes of up- or down-regulation at both timepoints. These genes include ENSG00000243679, ANKRD33, SMIM10, HNRNPA1L3, ARHGAP11B, ENSG00000273619, SNORA67 and POC1B-GALNT4 (**Supplementary Table S3**).

**Figure 3:**
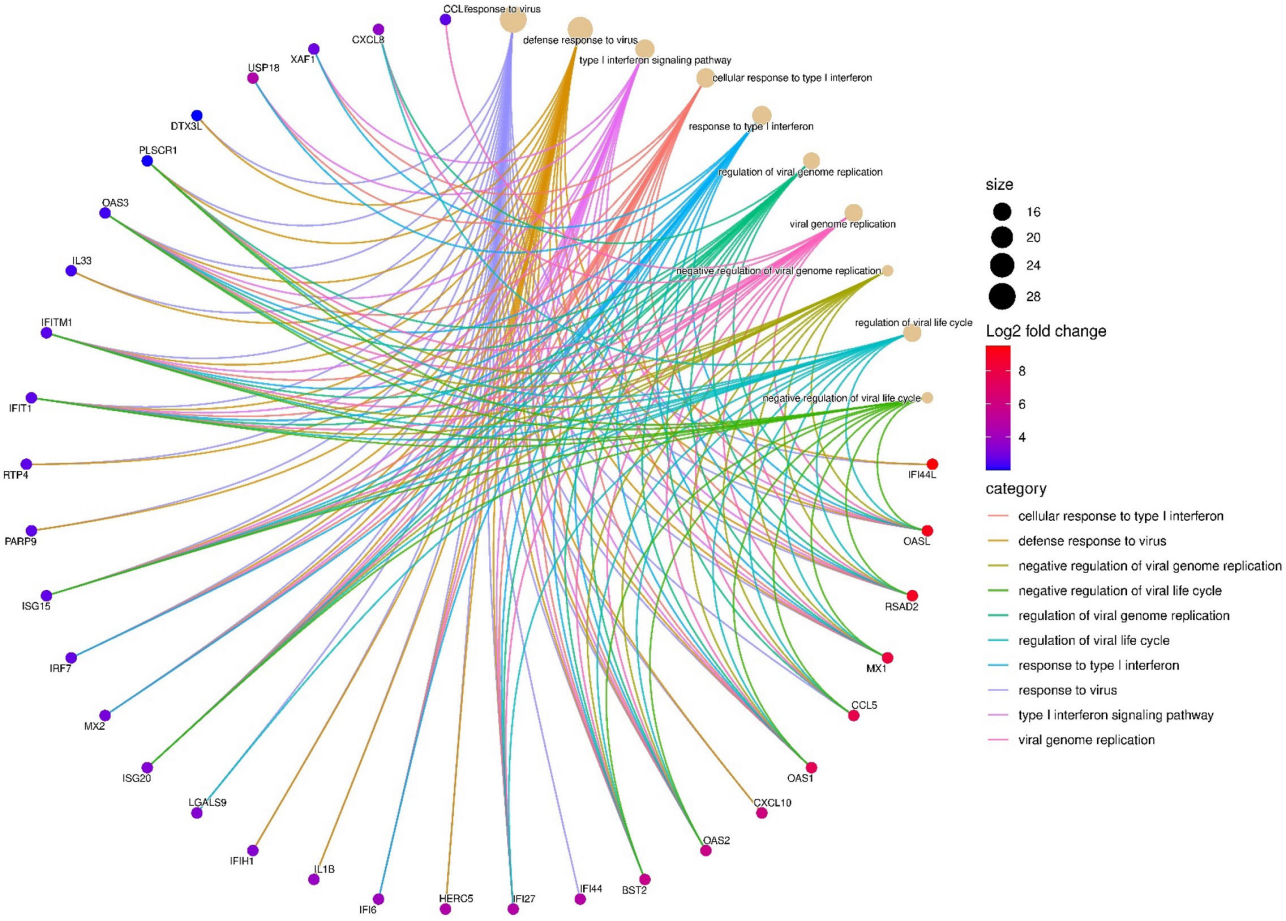
Top enriched host transcriptome biological processes (n=10) by category are shown at 24hpi, and key differentially expressed genes (DEGs) (n=32) involved in *C. trachomatis* E/Bour strain infected human primary endocervical fibroblast (ECS) cells relative to mock infected cells at 24hpi (FDR ≤0.05, log_2_FC ≥2.0). The most enriched pathway was “Response to Virus” with 28 DEGs. Gene enrichment analysis was visualized using SRplot (Tang et al., 2023).

**Figure 4:**
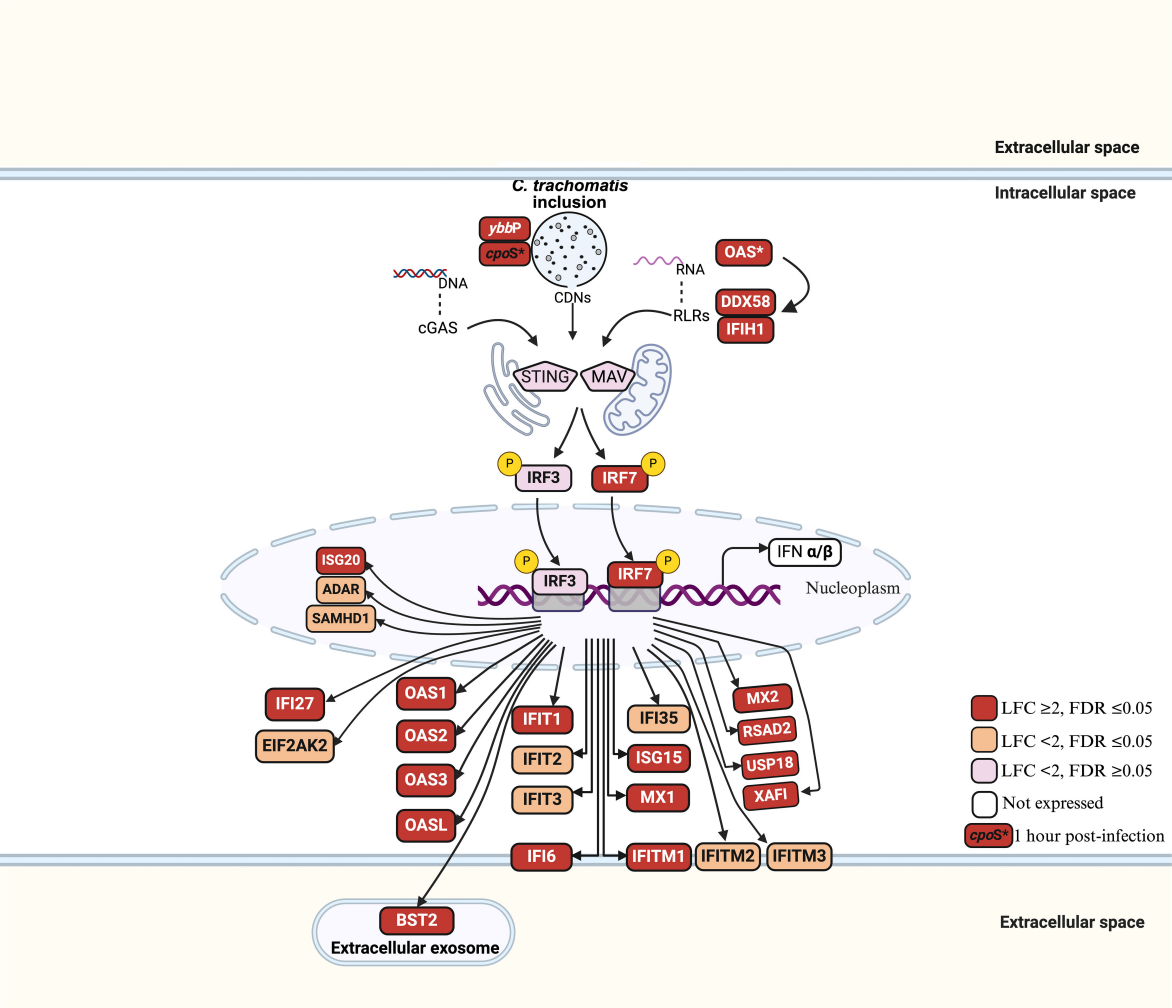
Host gene activation via cGAS-STING and RLR-MAV signaling pathways in *C. trachomatis* E/Bour-infected human primary endocervical fibroblast (ECS) cells at 24hpi. Red boxes: LFC ≥ 2, FDR ≤ 0.05 (significantly upregulated). Orange boxes: LFC < 2, FDR ≤ 0.05 (moderately upregulated). Pink boxes: LFC < 2, FDR > 0.05 (not significantly upregulated). Unfilled boxes: Genes not expressed. OAS*: OAS1, OAS2, OAS3 and OASL genes. P represents phosphorylation.

Gene set enrichment analysis of upregulated DEGs at 24hpi showed that the most enriched biological process (BP) term was “response to virus” with 28 genes. This occurred principally through genes participating in the cellular response to type I IFN (**Figure 3**; **Supplementary Table S4a**). A separate pathway enrichment analysis performed on all upregulated DEGs using the Reactome database showed IFN alpha(α)/beta(β) signaling as the most enriched pathway with the majority of the DEGs being IFN-stimulated genes (ISGs) (**Supplementary Table S5a**). Surprisingly, neither IFN-α nor IFN-β were expressed in these cells either at 1hpi or 24hpi (**Supplementary Table S5b**), suggesting that the upregulation of ISGs occurred in the absence of IFN-α and IFN-β. We therefore investigated the possible activation of the cyclic GMP–AMP synthase (cGAS)–stimulator of interferon genes (STING) and retinoic acid-inducible gene I (RIG-I)-like receptors (RLRs)-MAV pathways for host recognition of cytosolic chlamydial DNA and RNA, respectively. Seven key genes participating in the upstream processes of the cGAS-STING and RLR-MAV pathways were significantly upregulated without the production of detectable IFN-α and IFN-β mRNA (**Figure 4**). These genes were DDX58, IFIH1, IRF7, and the OAS family members OAS1–3 and OASL. In total, we identified 19 upregulated DEGs and an additional eight upregulated genes with LFC of <2.0 (FDR ≤ 0.05) participating in the cGAS-STING and RLR-MAV pathways (**Figure 4**).

The BP term “response to molecule (lipopolysaccharide) of bacterial origin” was significantly enriched at 24hpi (**Supplementary Table S4a**). Of the 411 genes that are known to as participate in this term, 241 were expressed in ECS cells at 24hpi of which 68 met the FDR ≥0.05 criterion. Of these 68 DEGs, 10 were downregulated while 62 were upregulated. By further applying the LFC of ≥2.0, we identified 15 genes that were differentially expressed, and all of them were significantly upregulated. These genes included CCL5, CMPK2, CXCL10, CXCL2, PTGS2, IL1B, FOS, CXCL8, LGALS9, IL24, CCL2, CSF2, PLSCR1, NCF2 and IRAK2 (**Supplementary Table S4b**). Of note, five of these upregulated genes (CCL2, CCL5, CSF2, CXCL10, CXCL2) are involved in leukocyte recruitment and activation.

### *C. trachomatis* transcriptome during infection of primary endocervical cells

Analyses of the *Ct* bacterial transcriptome at 1hpi and 24hpi provides an opportunity to understand host-pathogen interactions within the same host cell infection system. Differential gene expression analyses at these two timepoints in primary cells can aid in discerning mechanisms of *Ct* pathogenesis that are more relevant for human infection.

**Figure 5:**
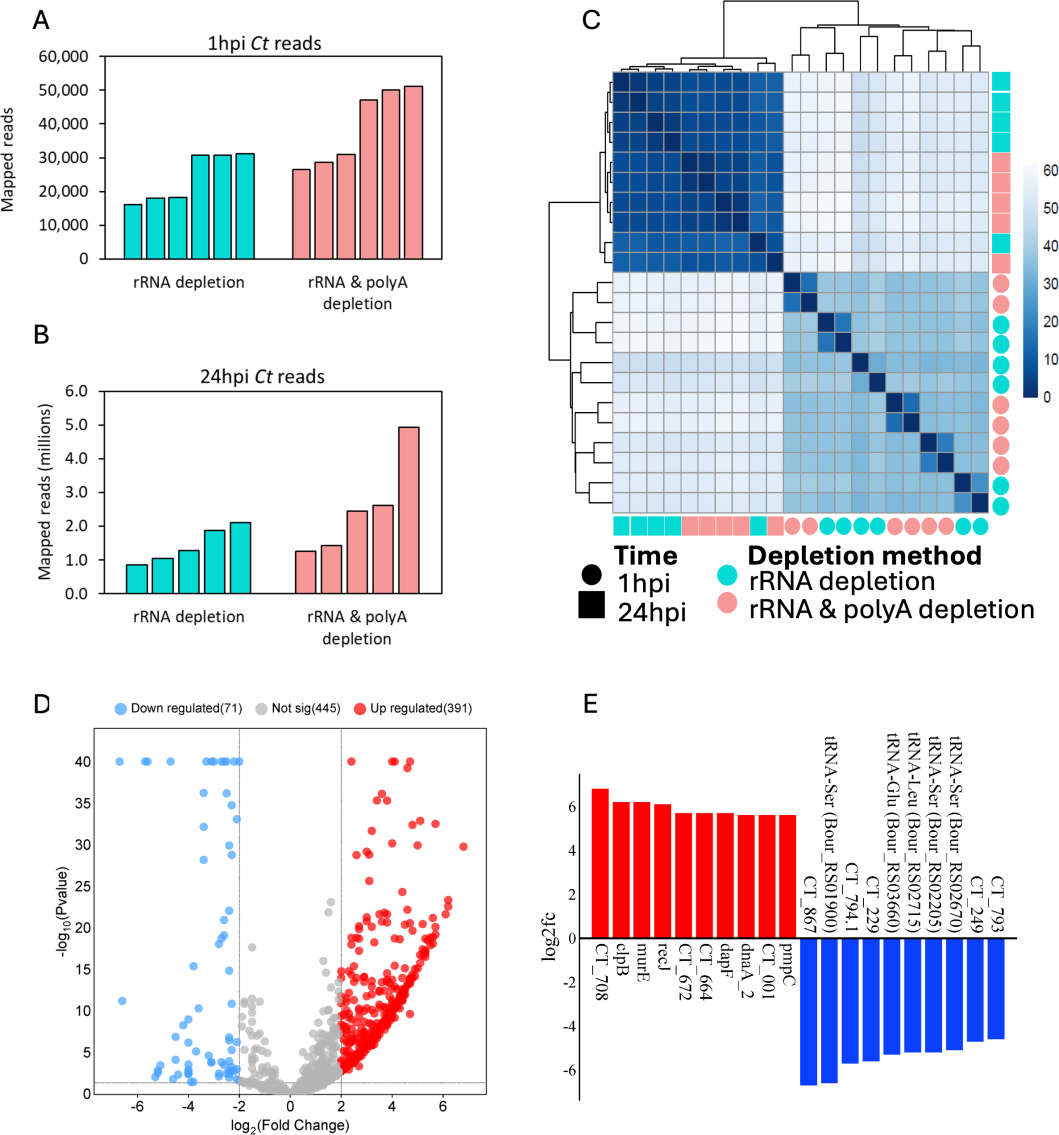
*C*. *trachomatis* (*Ct*) differentially expressed genes (DEGs) and mapped reads for reference strain E/Bour infected human primary endocervical fibroblast (ECS) cells. Uniquely mapped *Ct* reads from both rRNA depletion and rRNA plus polyA depletion methods are shown for **(A)** 1hpi and **(B)** 24hpi. **(C)** Euclidean distance between variance stabilized *Ct* read counts showing both timepoints and depletion methods. **(D)** Volcano plot showing *Ct* DEGs between 24hpi relative to 1hpi (FDR ≤0.05, log_2_FC ≥2.0). Blue dots (downregulated), higher expression at 1hpi relative to 24hpi; red dots (upregulated), higher expression at 24hpi relative to 1hpi; grey dots, no significant difference. **(E)** Top differentially expressed *Ct* genes at 24hpi (red, n=10) relative to 1hpi (Blue, n=10).

To adequately capture *Ct* transcripts, two depletion methods were utilized for the RNA samples obtained from each timepoint: a) rRNA depletion only; used to study both *Ct* and host transcriptome profiles; and b) rRNA depletion with polyA depletion; used to study *Ct* transcriptome profiles only. At 1hpi, the rRNA depletion method generated an average of 24,191 *Ct* reads per sample (total = 145,143), while rRNA and polyA depletion generated an average of 39,083 *Ct* reads per sample (total = 234,500) (**Figure 5a**). At 24 hpi *Ct* reads from the rRNA depletion method averaged 1,429,227 reads per sample (total = 7,146,133), while rRNA and polyA depletion averaged 2,533,238 reads per sample (total = 12,666,188) (**Figure 5b**). A Euclidean distance between variance stabilized read count for all samples was computed, and the resulting clustering showed a distinct clustering between 1hpi and 24hpi *Ct* reads (**Figure 5c**). Taken together, a total of 379,643 and 19,812,321 *Ct* reads were available at 1hpi and 24hpi respectively for differential expression analysis. At 1hpi, *Ct* genome coverage ranged from 4.7x to 14.8x (mean: 9.1x; standard deviation: 3.5), while at 24hpi, coverage ranged from 243.8x to 1419.6x (mean: 712.6x; standard deviation: 343.9).

Using a minimum count per million of 0.5 in at least 5 samples, a total of 338 *Ct* genes were identified as expressed at 1hpi representing about a third of the known *Ct* transcriptome. To identify genes with high expression levels, the minimum count per million threshold was increased to 100. With this cut-off, 184 genes were found to be highly expressed at 1hpi (**Supplementary Table S7a**). The top 10 highly expressed early genes were *ompA*, encoding the most abundant outer membrane protein; *npt1*, encoding an ATP/ADP exchange transporter involved in host energy parasitism; CT147, encoding an inclusion membrane protein likely involved in avoiding fusion with host lysosomes; CT529, encoding a putative inclusion membrane protein; *cpoS* (CT229), encoding an inclusion membrane protein important in host trafficking system modification; *omcB*, encoding the conserved outer membrane complex protein; *rpo*B and *rpo*C, encoding DNA-directed RNA polymerase subunits; *fusA*, encoding elongation factor G; and *ahpC*, encoding an antioxidant thiol-specific peroxidase. A complete list of the highly expressed early genes is shown in **Supplementary Table S7a**.

Using the same thresholds as 1hpi, 914 genes were expressed at 24hpi, of which 632 were found to be highly expressed. Not surprisingly, four early genes were also among the top 10 highly expressed genes at 24hpi as they are primarily constitutively expressed (*ompA, rpoB, rpoC* and *fusA*). Other members of the top 10 highly expressed genes were *nrdA*, a gene encoding a ribonucleoside-diphosphate reductase subunit alpha that catalyzes the biosynthesis of deoxyribonucleotides; *glgA*, a glycogen synthase gene; CT691, a TIGR00153 family protein gene; *rpsA*, a gene encoding the 30S ribosomal protein S1; *ndk*: a nucleoside-diphosphate kinase gene; and CT708 that encodes the DEAD/DEAH box helicase (**Supplementary Table S7b**).

When both timepoints were compared with the cut-off criteria of FDR ≤0.05, Log_2_FC ≥2.0, and CPM ≥0.5, 462 DEGs were identified (**Figure 5d**). These included 71 genes that were highly expressed at 1hpi, and 393 genes at 24hpi. Top DEGs at 1hpi include CT867, a gene encoding an effector protein with deubiquitinase enzyme activity; *cpoS* (CT229); and the inclusion (*inc*) gene CT249 (**Figure 5e**). Of note, while only 11 tRNA genes were differentially expressed at 1hpi relative to 24hpi, they made up 50% (10/20) of the top 20 DEGs. These tRNA are tRNA-Ser (3 copies), tRNA-Glu, tRNA-Leu (2 copies), tRNA-Val, tRNA-Arg, tRNA-Gln, tRNA-Asp, and tRNA-Phe (**Supplementary Table S6**). At 24hpi, the top DEGs include *recJ*, a DNA specific exonuclease gene; *pmpC*, a polymorphic outer membrane protein (Pmp) gene; and CT708 (**Figure 5e**). We also identified 60 DEGs that were hypothetical proteins (**Supplementary Table S6**).

**Figure 6:**
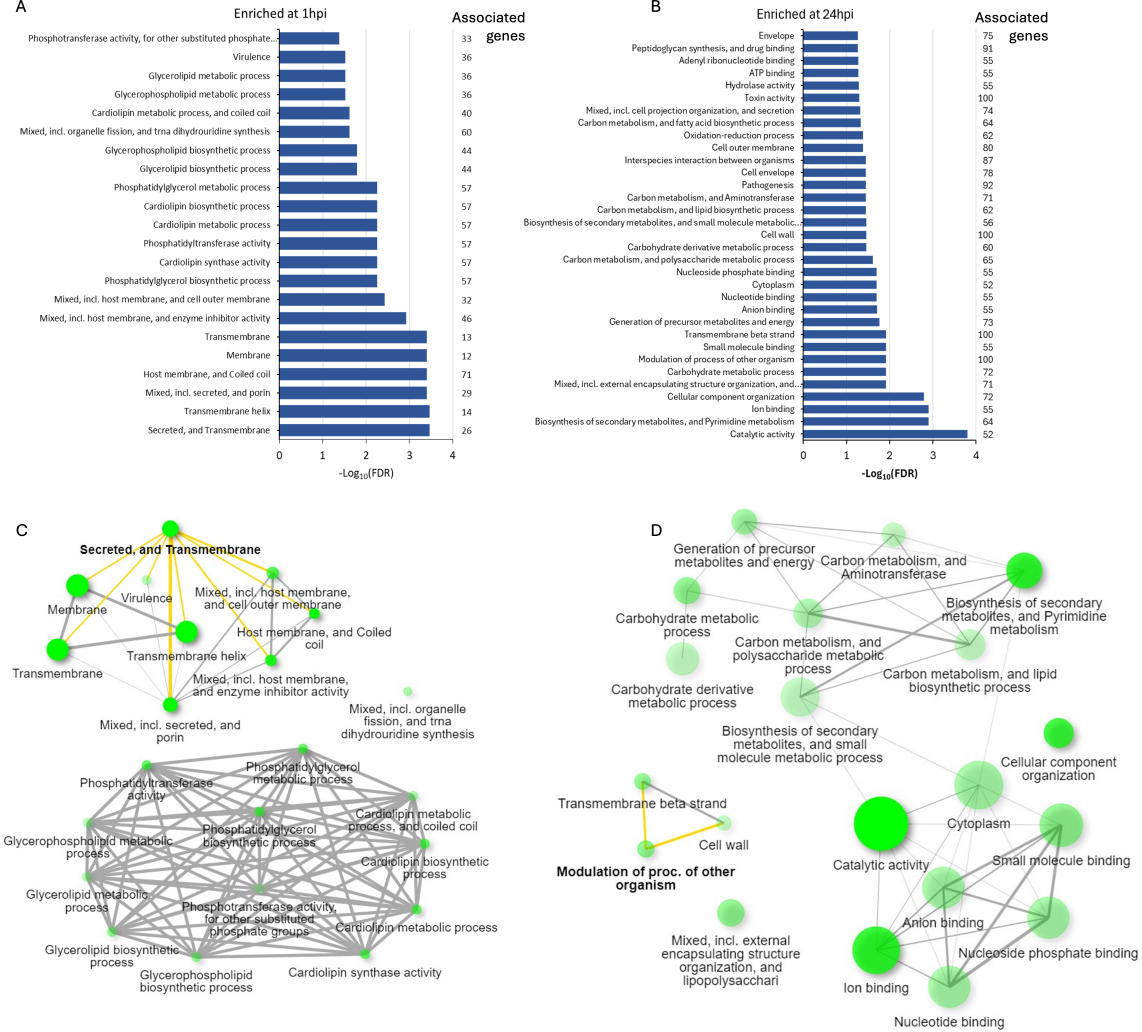
Enriched pathways identified by gene set enrichment analysis for the differentially expressed *C*. *trachomatis* genes at **(A)** 1hpi and **(B)** 24hpi showing the number of genes associated with each enriched term. Network interaction of key pathways and terms present at **(C)** 1hpi and **(D)** 24hpi with key terms further explored in this study highlighted in yellow. The color intensity of the green circles corresponds to the enrichment scores while the size corresponds to the number of DEGs identified in each pathway.

Gene set enrichment analysis of *Ct* DEGs with higher expression at 1hpi showed “Secreted and Transmembrane” as the most enriched term (**Figure 6a**; **Supplementary Table S8a**). DEGs in this term included seven inclusion membrane protein genes (*incB, incC, incD, incE, cpoS*, CT227, and CT228). A manual search of the literature showed a total of 30 *inc* genes were differentially expressed in this study, 21 (68%) of which had a significantly higher expression level at 1hpi relative to 24hpi (**Table 1**). Non-inclusion proteins (n=9) known to be secreted into the host cytoplasm were found to be differentially expressed in this study. Of note, only three of these had a significantly higher expression at 1hpi (CT473: a hypothetical protein gene; CT867 and CT868: deubiquitinase genes) (**Table 1**).

**Table 1:** Differential expression of *C. trachomatis* inclusion (*inc*) and non-inclusion membrane genes at 24hpi relative to 1hpi in human primary endocervical fibroblast (ECS) cells infected with reference strain E/Bour.

Gene ID (name)	24hpi Fold change (Log_2_)	Gene ID (name)	24hpi Fold change (Log_2_)
CT229 (*cpoS*)	-5.6	CT556	2.0
CT249	-4.7	CT565	2.0
CT357	-4.2	CT225	2.3
CT196	-4.0	CT484	2.6
CT233 (*incC*)	-3.8	CT223 (*ipaM*)	3.1
CT440	-3.7	CT642	3.2
CT232 (*incB*)	-3.6	CT616	3.8
CT018	-3.4	CT728	4.0
CT116 (*incE*)	-3.1	CT850	4.2
CT147	-2.7		
CT789	-2.7	^#^CT867 (deubiquitinase gene)	-6.7
CT228	-2.6	^#^CT868 (deubiquitinase gene)	-3.4
CT135	-2.5	^#^CT473	-2.5
CT483	-2.4	^#^CT711	2.7
CT115 (*incD*)	-2.4	^#^CT858	2.8
CT788	-2.4	^#^CT737	2.8
CT036	-2.3	^#^CT694	2.9
CT227	-2.3	^#^CT049	3.0
CT358	-2.1	^#^CT695	3.4
CT134	-2.1	^#^CT621	5.3
CT005	-2.1		

Boxed genes, non-inclusion protein genes; Blue font, higher expression at 1hpi relative to 24hpi; Red font, higher expression at 24hpi relative to 1hpi.

Gene set enrichment analysis of *Ct* DEGs with higher expression at 24hpi revealed “catalytic activity” as the most enriched term (**Figures 6b, d**). Other enriched terms include “ion binding,” “carbohydrate metabolic process,” and “modulation of process of other organisms” (**Supplementary Table S8b**). As “modulation of process of other organisms” was the highest enriched term with 100% of its associated genes differentially expressed, we investigated this process further. Of the 11 DEGs identified in this process, eight were polymorphic membrane protein (*pmp*) genes: *pmpB* to *pmpI*. The other three were hemolysin family protein genes (CT256, CT257 and CT423).

## Discussion

An understanding of the complex host-pathogen interactions between the human host and *Ct*, especially in the early stages of infection, has the potential to aid in identifying biomarkers and develop diagnostic tools and therapeutic targets for preventive strategies. Previous attempts to study transcriptome-wide host-*Ct* interactions utilized either microarray or RNA-seq technologies and lab-adapted immortalized cells such as HeLa229 and HEp2 (Belland et al., 2003b; Humphrys et al., 2013). While immortalized cells are excellent models and provide an opportunity for hypothesis generation/testing, they are unlikely to represent the actual *in vivo* response to the pathogen. To date, no transcriptome-wide analysis of primary human cell responses to *Ct* infection and the simultaneous responses of *Ct* to those cells have been reported. This study is therefore the first to describe the transcriptional profiling of *Ct*—in particular strain E that represents the most prevalent strain responsible for STIs in women—and host cells in an *ex vivo* human primary cell culture system. We show that clinically relevant data that are essential for interrogating *Ct* pathogenesis are obtainable using this model.

Previous work describing the early host transcriptome following *Ct* infection reported differential regulation of 13 host genes in HeLa cells infected with *Ct* strain L_2_/434/Bu at 2hpi using microarray technology (Xia et al., 2003), and 622 host genes in Hep2 cells infected with E/Bour at 1hpi based on dual RNA-seq (Humphrys et al., 2013). In the current study, 168 host genes were differentially expressed in E/Bour infected ECS cells at 1hpi, 107 of which were upregulated while 67 were downregulated, representing 3.7x fewer DEGs compared to the prior study (Humphrys et al., 2013). We suspect that the lower number of DEGs in our study points to a less aggressive primary cell response to *Ct* infection compared to immortalized cells. This would be consistent with the stronger upregulation of extracellular matrix remodeling in immortalized cells (Humphrys et al., 2013) compared to primary cells.

The top host upregulated gene at 1hpi was SNORD3D that encodes a member of the box C/D small nucleolar ribonucleoproteins (snoRNPs). These proteins catalyze site-directed modifications, typically the 2′-O-methylation of ribosomal RNA (Baldini et al., 2021). While their role in host immune responses are not well known, a recent study showed that increased box C/D snoRNPs corresponded to a repressed immune function in the *Caenorhabditis elegans* model of *Pseudomonas aeruginosa* infection (Tjahjono et al., 2022). In *Ct*, SNORD3D upregulation suggests that the organism may influence host RNA processing and ribosome biogenesis to suppress host innate immune responses and thereby evade detection. Box C/D snoRNPs maintain mitochondrial dynamics that include function and surveillance (Tjahjono et al., 2022). *Ct* manipulates mitochondrial fusion to regulate host cell metabolism and acquire ATP and control mitochondrial fission to prevent apoptosis (Yang et al., 2022), both of which are essential for *Ct* growth/reproduction and survival, respectively. However, the lack of specific studies on the role of box C/D snoRNPs in chlamydial pathogenesis highlights the need for further research.

In addition to multiple novel protein genes and pseudogenes, we found several host DEGs at 1hpi that were lincRNAs. The biological role of most lincRNAs are largely unknown, however, some have been shown to participate in either negative or positive modulation of host responses to bacterial infection (Agliano et al., 2019; Arunima et al., 2023). Recent data show that lincRNAs may promote *Ct* replication *in vitro* (reviewed in (Arunima et al., 2023)). However, none of the nine lincRNAs identified in the current study have been studied in the context of *Ct* pathogenesis. The absence of functional characterization for many of these lincRNAs emphasizes the need for future studies to determine their precise contributions to *Ct* infection dynamics, particularly their potential as biomarkers or therapeutic targets.

At 24hpi, the host transcriptome primarily represented an immunoreactive phenotype accompanied by upregulated intracellular signaling. Alongside pro-inflammatory cytokines and adhesion molecules, the immune response was characterized by strong upregulation of genes linked to IFNα and IFNβ and, to a lesser extent, the IFNγ signaling responses. Since none of the IFNs were differentially expressed in infected cells, it is possible that *Ct*-related CDNs and/or RNA/DNA may be sufficient to trigger an IFN signaling cascade with a bias towards type I IFNs. The role of type I IFN signaling in the clearance of bacteria is enigmatic as both IFNα and IFNβ seem to have opposite effects (McNab et al., 2015; Boxx and Cheng, 2016). A recent study showed that type I IFNs did not prevent infection or replication of *Ct* in primary synovial fibroblasts (Di Pietro et al., 2020) similar to our findings. A separate study showed that type I IFNs may work in concert with the type II IFN response to inhibit *Ct* growth in epithelial cells (Ishihara et al., 2005). However, depending on the levels of type I IFNs, B cells may be inhibited along with reducing macrophage responses to IFNγ thereby facilitating infection (McNab et al., 2015). While it is evident that *Ct* does not completely evade immune responses, it remains unclear if the induction of a strong type I IFN response by ECS cells at 24hpi is sufficient to clear infection, facilitate infection, or encourage persistence by regulating indoleamine 2,3-dioxygenase (IDO), the induction of which depletes tryptophan, an essential amino acid required for *Ct* growth and replication (Puccetti, 2007; Trinchieri, 2010; Bommana et al., 2021).

The cGAS-STING and RLRs-MAV pathways are important for the recognition of cytosolic chlamydial DNA and RNA, respectively, by host cells (Wen and Li, 2020) and typically end in the production of type I IFN genes and autophagy. The *Ct ybbP* gene has been shown to be crucial in the synthesis of cyclic di-AMP, a major ligand in the cGAS-STING pathway (Barker et al., 2013; Meier et al., 2023), and was upregulated 16-fold at 24hpi (**Supplementary Table S6**). However, neither IFNα nor IFNβ were expressed at 24hpi by the primary cells. Surprisingly, a strong stimulation of multiple type I IFN-stimulated genes (ISGs) was observed. There is evidence for cooperation between the cGAS-STING and RLRs-MAV pathways for ISG production (Zevini et al., 2017). Data from the current study suggests that at 24hpi, the main response of primary cells to *Ct* was the induction of ISGs through the cGAS-STING and RLR pathways (**Figure 4**). The lack of detectable type I IFN gene expression is intriguing and might suggest a deliberate evasion strategy. *Ct* may inhibit upstream IFN production to prevent a full-scale immune response, while allowing ISG activation to maintain host cell viability and metabolic activity. This selective modulation could also help *Ct* evade inflammatory cell death pathways that are typically triggered by robust IFN responses.

A recent study using a murine L929 immortalized fibroblast cell line infected with *Ct* strain L_2_ reported the expression of over 700 *Ct* genes as early as 1hpi, suggesting widespread early transcriptional activity (Wurihan et al., 2024). In contrast, our study identified 338 *Ct* transcripts at 1hpi in ECS cells infected with the E/Bour strain. Several key methodological differences likely contribute to this discrepancy. Notably, the previous study employed a high MOI of 50, whereas we employed an MOI of 1 to better reflect physiologically relevant infection burden. While a high MOI may not accelerate developmental timing (Chiarelli et al., 2020), it may influence the number of infected cells resulting in enhanced transcript detection. Additionally, differences in host cell type (i.e., immortalized versus primary cells) as well as *Ct* strain further influence early gene expression profiles as is evident in our study. Together, these factors highlight the importance of infection context in interpreting pathogen transcriptional dynamics.

Inclusion membrane proteins are a known group of *Ct* proteins that mediate *Ct*-host interactions (reviewed in (Bugalhão and Mota, 2019)). For instance, *incD* redirects host lipids away from their intended host destinations and towards the chlamydial inclusion (Derré et al., 2011; Agaisse and Derré, 2014), while *cpoS* (CT229) controls host cell vesicular trafficking (Bugalhão and Mota, 2019). In the current study, we report the upregulation of 21 known *inc* genes at 1hpi compared to 24hpi in primary cells (**Table 1**). In contrast, only 9 *inc* genes had higher expression levels at 24hpi compared to 1hpi. While the functions of the majority of *inc* genes identified in this study are still unknown, we suspect that they are crucial to a successful infection event and subsequent establishment of the *Ct* inclusion in primary host cells.

This study also underscores the complementary roles of upregulated hemolysin-like proteins (encoded by CT256, CT257, CT423) and PmpB to PmpI in the pathogenesis of *Ct* during the mid-infection stage (24hpi). Given their known roles in other bacteria, hemolysin-like proteins likely facilitate *Ct* survival by modifying the host intracellular environment, potentially through membrane disruption, nutrient acquisition, and suppression of immune responses (Aroian and van der Goot, 2007; Gonzalez et al., 2011; Wiles and Mulvey, 2013; Mondal and Chattopadhyay, 2022). In parallel, PmpB to PmpI are critical for mediating host-pathogen interactions such as adhesion, intracellular trafficking, and immune evasion (Vasilevsky et al., 2016; Pickering et al., 2017; Jury et al., 2023). Their concurrent expression suggests a coordinated strategy where hemolysin-like proteins create conditions favorable for Pmp-mediated processes. For instance, hemolysins may alter autophagy or inhibit lysosomal degradation, enabling Pmps to enhance *Ct* adhesion, endosomal trafficking and inclusion stability as well as other interactions with host cellular pathways. Both groups appear to contribute to *Ct*’s ability to evade host immune defenses while ensuring access to resources needed for replication. Functional studies investigating the co-regulation and physical interactions of these proteins could provide insights into their mechanistic roles and reveal potential targets for therapeutic intervention.

We also identified 60 DEGs that were hypothetical proteins with unknown functions. This observation suggests that the transcriptome of *Ct* at the early infection stage is currently under-characterized thus hampering an adequate understanding of their roles in *Ct* pathogenesis. Describing the role of these genes and their proteins may provide more insights into the natural course of *Ct* pathogenesis.

This study offers novel insights into host and *Ct* transcriptional dynamics in ECS cells, although some limitations should be noted. Similar to human immortalized cells that are derived from a single donor, our ECS cells were obtained from a single donor. Further transcriptomic analyses in ECS cells from additional donors are warranted to expand on our findings and capture any inter-individual variations. Further, while we previously performed extensive head-to-head comparisons between *Ct* infected primary and immortalized cells that informed our experimental approach and framework for interpreting our findings (Jolly et al., 2019), we did not do so here. These types of experiments could be performed to directly compare specific cell-type responses. Finally, protein-level quantitative assays on cell culture supernatants would be valuable to further refine and substantiate the transcriptomic data.

In conclusion, our study highlights the utility of *ex vivo* human primary cell cultures and dual RNA-seq in uncovering unique host-pathogen dynamics during *Ct* infection. Early infection showed a subdued host response alongside elevated expression of *Ct* inclusion genes, while mid-infection revealed robust host immune activation, including upregulated interferon-stimulated genes and cGAS-STING and RLR pathway activation. On the bacterial side, mid-stage expression emphasized hemolysin-like proteins and Pmps. These findings underscore the value of physiologically relevant *ex vivo* models for studying *Ct* pathogenesis that include the use of clinically relevant *Ct* strains and identifying novel therapeutic targets. Future research should explore the roles of uncharacterized genes and validate these results *in vivo* to deepen our understanding of *Ct* biology, pathogenesis, and host responses.

## Data Availability

The datasets presented in this study can be found in online repositories. The names of the repository/repositories and accession number(s) can be found in the article/**Supplementary Material**.
