# Supplementary material for: Transcriptional profiling of Chlamydia trachomatis and its host in an ex vivo endocervical primary cell culture system using dual RNA sequencing
Source: Front Cell Infect Microbiol. 2025 Jun 17;15:1613922. doi: 10.3389/fcimb.2025.1613922 (PMC12209195; doi:10.3389/fcimb.2025.1613922)
Supplement: Supplementary file 2 [file DataSheet1.pdf]

Transcriptional profiling of *Chlamydia trachomatis* and its host in an *ex vivo* endocervical primary cell culture system using dual RNA sequencing

Olusola Olagoke[a]*, Siddharth Chittaranjan[a]*, and Deborah Dean[a,b,c,d†]

[a]Departments of Medicine and Pediatrics, University of California San Francisco, Oakland, CA, USA

[b]Department of Bioengineering, University of California San Francisco and University of California Berkeley, San Francisco, CA, USA

[c]Bixby Center for Global Reproductive Health, University of California San Francisco, San Francisco, CA, USA

[d]Benioff Center for Microbiome Medicine, University of California San Francisco, San Francisco, CA, USA

Supplementary Figure S1

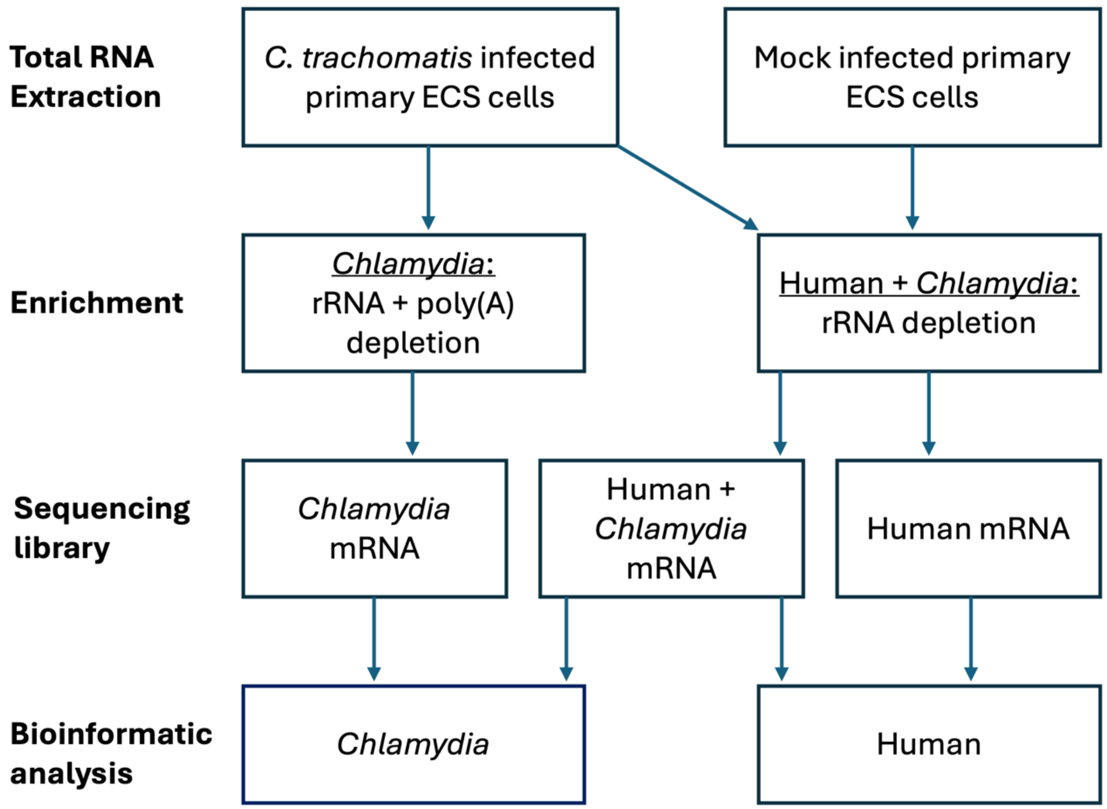

Figure S1: Simplified workflow detailing the laboratory pipeline for RNA extraction and enrichment as well as library sequencing.
